# Triapine Radiochemotherapy in Advanced Stage Cervical Cancer

**DOI:** 10.3389/fonc.2018.00149

**Published:** 2018-05-07

**Authors:** Charles A. Kunos, S. Percy Ivy

**Affiliations:** Cancer Therapy Evaluation Program, National Cancer Institute, Bethesda, MD, United States

**Keywords:** triapine, cervical cancer, uterine cervix cancer, vaginal cancer, radiation therapy, cisplatin

## Abstract

Clinical ribonucleotide reductase (RNR) inhibitors have reinvigorated enthusiasm for radiochemotherapy treatment of patients with regionally advanced stage cervical cancers. About two-thirds of patients outlive their cervical cancer (1), even though up to half of their tumors retain residual microscopic disease (2). The National Cancer Institute Cancer Therapy Evaluation Program conducted two prospective trials of triapine–cisplatin–radiation to improve upon this finding by precisely targeting cervical cancer’s overactive RNR. Triapine’s potent inactivation of RNR arrests cells at the G1/S cell cycle restriction checkpoint and enhances cisplatin–radiation cytotoxicity. In this article, we provide perspective on challenges encountered in and future potential of clinical development of a triapine–cisplatin–radiation combination for patients with regionally advanced cervical cancer. New trial results and review presented here suggest that a triapine–cisplatin–radiation combination may offer molecular cell cycle target control to maximize damage in cancers and to minimize injury to normal cells. A randomized trial now accrues patients with regionally advanced stage cervical cancer to evaluate triapine’s contribution to clinical benefit after cisplatin–radiation (clinicaltrials.gov, NCT02466971).

## Introduction

Cervical cancer forecasts as the fourth most common any-type cancer in women worldwide in 2018 (3). And it remains the fourth leading cause of cancer-related death (3). About 36% of new cases in American women are staged as regionally advanced at first diagnosis (4). This means that their disease is confined in the cervix or nearby organs or lymph nodes (International Federation of Gynecology and Obstetrics stage IB2 to IVA). Patients with this stage of disease undergo once weekly cisplatin chemotherapy (40 mg m^−2^) and daily radiation (180 cGy per Monday to Friday) repeated for 5 weeks followed by intracavitary brachytherapy (1, 5). A 60-month (5-year) survival rate for such treated patients is 60% (1).

**Figure 1:**
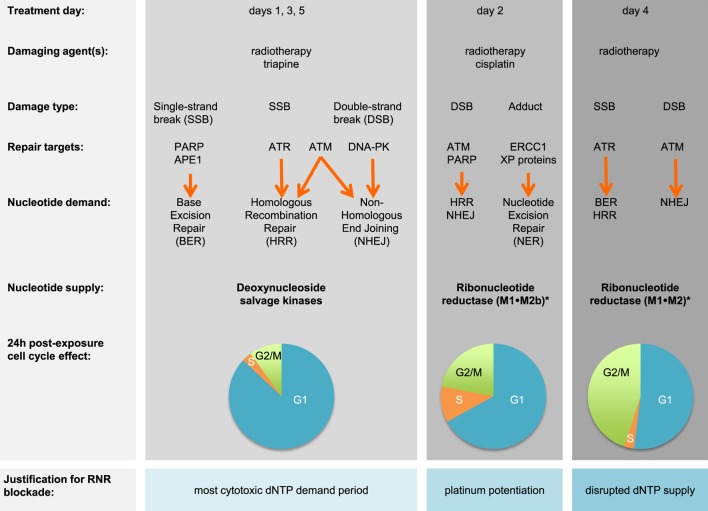
Triapine–cisplatin–radiation treatment and cell cycle targets. Damaging agents and DNA damage response repair targets are charted in relation to triapine–cisplatin–radiation treatment. Shown in bold are nucleotide supply chain elements likely to be active. *Data suggest M2 or M2b recycling after triapine exposure occurs over an 18-h period, but further validation is needed (21). Pie charts indicate representative 24-h cell cycle effects (22). Abbreviations: APE1, AP endonuclease 1; ATM, ataxia-telangiectasia mutated; ATR, ataxia-telangiectasia and Rad3-related; DNA-PK, DNA-dependent protein kinase; dNTP, deoxynucleotide triphosphate; ERCC1, DNA excision repair protein 1; PARP, poly(ADP-ribose) polymerase; RNR, ribonucleotide reductase; XP, xeroderma pigmentosum.

Prognostic factors such as cell type, histological grade, and invasiveness provide only a partial explanation of why only 6 in 10 survive after cisplatin–radiation (6). An overactive DNA damage response that involves ribonucleotide reductase (RNR) might further explain this clinical result (7, 8). RNR substitutes a hydroxyl group in a ribonucleotide diphosphate for hydrogen for its corresponding deoxyribonucleoside diphosphate (dNDP) that ultimately can be used in DNA duplication or its repair (9). Both of its higher order (α_6_β_6_ or α_6_β_2_) forms are active (10). RNR’s large subunit α (M1) contains: (1) a catalytic pocket for rNDP substrates; (2) a specificity site that controls which nucleotides are made; and (3) an activity site that regulates its own activity through biologic feedback (11). Its catalytic pocket is the drug target for gemcitabine (12). RNR’s small subunit β (M2 or M2b) shuttles a critical diferric tyrosyl radical to the M1 subunit’s catalytic site (13). The free radical is the drug target for hydroxyurea or triapine (14, 15). The response to DNA damage will be different depending on cell cycle status (Figure 1). M1 is long-lived and found in all cell cycle phases (16). M2 has a KEN-box sequence recognized by the Cdh1-anaphase-promoting complex that degrades it in late mitosis (17, 18). M2b lacks the KEN-box and thus can be detected in all cell cycle phases. M2b transcription is p53 dependent (19). This means that cancers in G1-phase of the cell cycle do not have M2 available to pair up with M1 for active enzyme, and therefore, must depend upon M2b to be available for active enzyme. When a drug like triapine inactivates RNR, deoxynucleoside salvage kinases supply DNA precursors for duplication or repair (20).

Triapine (3-aminopyridine-2-carboxaldehyde thiosemicarbazone) potently blocks RNR activity (15). But triapine as monotherapy has been ineffective (0–7% response rate) in the clinic when given up to 96 mg m^−2^ daily (23–25). A triapine–cisplatin combination was tolerable but also clinically ineffective with no observed responses (26). A triapine–cisplatin–paclitaxel combination too was tolerable but found to not result in objective responses (27). Cancer Therapy Evaluation Program (CTEP) repositioned triapine as a radiation modifier in 2006. Early experiments showed that confluent and growth-arrested cervical cancer cells had a 17-fold rise in RNR M2 expression about 18 h after radiation exposure (28). There was also a fourfold increase in their RNR dNDP output (or DNA precursor output) about 24 h after radiation exposure (28). Later experiments reinforced the notion that triapine offers molecular target control of RNR activity for up to 18 h until M2 expression restores enzyme output (29). It was shown that triapine strongly arrested cells at a G1/S-phase cell cycle restriction checkpoint for up to 18 h, left radiation-induced DNA damage unrepaired for at least 6 h, and profoundly sensitized cancers to radiation–cisplatin cytotoxicity (20–22, 30).

This article provides a state-of-the-art perspective on a triapine–cisplatin–radiation combination for the treatment of regionally advanced stage cervical cancer. The first trial (#7336) reviewed here was a phase I dose-finding safety study (clinicaltrials.gov, NCT00335998) (31). The second trial (#8327) was a single-arm phase II efficacy study (clinicaltrials.gov, NCT00941070) (32). Now that the median survivor follow-up in the two trials exceeds 6 years, CTEP offers perspective on the challenges encountered and opportunities gained for clinical development of triapine as an anticancer agent.

## Challenges and Opportunities

Cancer Therapy Evaluation Program’s studies activated as a phase I trial in 2006 (#7336) and then a phase II trial in 2009 (#8327). The first trial recruited patients mainly with cervical cancers of any stage [10 (91%) of 11] (31). The second trial accrued any stage cervical or vaginal cancers (32). Tumor stage heterogeneity has posed a challenge to long-term interpretation of front-line triapine impact upon established cisplatin–radiation combination treatment. First, in CTEP trials, any cervical cancer disease lying outside a pelvic radiotherapy treatment beam portal was not irradiated, negating any possibility of radiation–triapine synergistic cytotoxicity. This too means that those front-line patients with initial extrapelvic or metastatic disease only had their unirradiated disease treated by cisplatin–triapine, which may have only modest anticancer activity in cervical cancer patients (26). In this article, we assessed long-term patient outcomes from those patients with only regionally advanced stage cervical (IB2 to IVA) or vaginal (II to IVA) cancers recruited in its trials. Patients previously analyzed were excluded for this article if they had metastatic disease at treatment onset (*n* = 4), extrapelvic disease (*n* = 2), death due to protocol-unrelated iatrogenic Mallory–Weiss tear (*n* = 1), or if they consented but had no treatment (*n* = 1). Twenty-nine patients are thus discussed below.

Another challenge in front-line triapine clinical development has been its administration schedule (Table 1). In trials, radiotherapy involved 25-fraction (180 cGy) Monday through Friday four-field box radiation beginning on day 1. This was followed by low-dose-rate (LDR) brachytherapy in one or two implants (day 49 or 56 ± 3 days). Or, there was an option for high-dose-rate (HDR) brachytherapy in five implants (e.g., days 42, 45, 49, 52, and 56). Total treatment time was to be 56 ± 3 days. The total prescription was 8,000 cGy (LDR) or 7,500 cGy (HDR) or more as clinically indicated. Cisplatin (40 mg m^−2^ capped at 70 mg) was moved to a Tuesday intravenous infusion (day 2). There was a treating physician’s option for a sixth infusion on day 36 ± 3 days. Because of triapine’s 2-h half-life, triapine (25 mg m^−2^) intravenous infusions were scheduled three times per week beginning on day 1. An often-held perception is that the number of triapine infusions is cumbersome to patients. To correct this notion, there may be an opportunity to study the oral triapine formulation (33) in this patient population as proposed in CTEP protocol #9892.

**Table 1:** Triapine–cisplatin–radiation weekly^a^ treatment schedule.

Monday	Tuesday	Wednesday	Thursday	Friday	Saturday	Sunday
Triapine	Cisplatin	Triapine	–	Triapine	–	–
Pelvic radiation	Pelvic radiation	Pelvic radiation	Pelvic radiation	Pelvic radiation	–	–

*^a^Treatment began on Monday day 1, and repeated each week for a total of 5 weeks (i.e., days 1, 8, 15, 22, and 29)*.

From the CTEP perspective, its trials were the first to used ^18^F-fluorodeoxyglucose (FDG) positron emission tomography (PET) at baseline and 3 months after treatment completion for prognostic treatment response assessment. This third challenge necessitated evaluation of its FDG PET metabolic response criteria, as there is the possibility of overestimating metabolic complete or partial response due to a substantial radiation cytotoxicity effect. In the trials, cervical tumor and lymph node regions defined on baseline scan were drawn as regions of interest (ROI) that would then be assessed on a 3-month follow-up scan. Uptake measurements were made for mean and maximum tumor ROI counts per pixel per second (calibrated as MBq L^−1^). The original FDG PET metabolic response evaluation criteria are found elsewhere (34). Briefly, a metabolic complete response was defined as absence of abnormal FDG uptake at sites of abnormal FDG uptake noted on the baseline scan. Partial metabolic response was 15–25% reduction in tumor FDG uptake. Stable metabolic response ranged between less than 15% reduction or less than or equal to 25% gain in tumor FDG uptake. Progressive metabolic disease was labeled as greater than 25% gain in tumor FDG uptake or appearance of new FDG uptake in metastatic lesions. On CTEP’s trials in cervical cancer, a computed tomography scan was co-acquired for anatomic detail. To overcome challenges, there was an opportunity to apply a vetted ratio of 3-month post-treatment to baseline pre-treatment FDG uptake in cervical tumor. A benchmark threshold ratio of 0.33 was applied for mCR (35). In these two trials, the 0.33 ratio benchmark served well as prognostic indicator of best response. There is further possibility to evaluate the performance of the 0.33 ratio benchmark in CTEP’s current randomized trial (clinicaltrials.gov, NCT02466971).

## Perspectives on New Trial Findings

Original patient demographics and tumor characteristics are summarized elsewhere (31, 32). The median age of the combined patients in this 29-patient analysis was 57 years, ranging between 33 and 68 years. All patients were female. Self-identified race was white (72%) or American black (28%). Ten percent identified their ethnicity as Hispanic or Latino. Squamous cell carcinoma was the most common type of tumor histopathology (93%). The 29-patient data in this analysis have a cutoff date of October 24, 2017.

Triapine–cisplatin–radiation appears safe and tolerable. Updated results for adverse events on these trials reveal immediate grade 3 or 4 gastrointestinal (nausea 11%, diarrhea 8%, anorexia 3%, and dehydration 3%) or hematological toxicities (thrombocytopenia 20%, lymphocyte decrease 16%, and anemia 7%) to be generally mild, reversible, but attributable to the combination. Triapine has one adverse event of particular interest to CTEP and requires adverse event of special interest reporting—methemoglobinemia. Triapine interacts with Fe(2+) hemoglobin forming Fe(3+) methemoglobin that does not deliver oxygen. For context, Fe(2+) hemoglobin normally auto-oxidizes to inactive Fe(3+) methemoglobin at a rate of nearly 3% per day. A reductase system counterbalances this process to normally limits methemoglobin concentrations to less than 1%. Given repeated exposure to triapine during the course of cisplatin–radiation treatment in these trials, methemoglobinemia and symptoms of dyspnea were monitored by CTEP on both trials. Management guidelines for methemoglobinemia were included in each protocol and can be reviewed elsewhere (36). At a recommended phase II trial dose of triapine 25 mg m^−2^, methemoglobinemia peaked on average at 2%. As compared to pre-infusion baseline, this post-infusion proportion was on average a fivefold rise in methemoglobin. The trials collectively observed one (3%) rectovaginal fistula occurring 22 months after treatment. This patient underwent diverting colostomy and hyperbaric oxygen treatment to aid healing of the rectovaginal fistula. Late ureteral obstruction was not encountered. An increased emphasis on morbidity outcomes as a component of regulatory filing makes careful monitoring of adverse events critical on the randomized trial of the triapine–cisplatin–radiation combination.

Confirmed clinical complete responses after triapine–cisplatin–radiation treatment were 100% on these trials (29 of 29, Table 2). Confirmed mCR rate was 95% (21 of 22, Table 2). Median FDG PET uptake before treatment was 14.3 (range 6.9–32.1) in the 29 patients analyzed here. Median uptake after triapine–cisplatin–radiation treatment was 2.8 (range 1.1–6.4) for the same population. A median uptake ratio was, therefore, resulted at 0.19 (range 0.06–0.56). A single patient who did not achieve an uptake ratio of less than 0.33 had the only partial metabolic response after combination treatment. And this patient developed progressive disease in an intrapelvic lymph node 8 months after combination treatment.

**Table 2:** Responses^a^ of cervical tumor to indicated treatment.

	Triapine–cisplatin–radiation	Cisplatin–radiation	Cisplatin-alone	Radiation-alone
Cisplatin dose (frequency)	40 mg m^−2^ (x1 weekly)	30 mg m^−2^ (x1 weekly)	50 mg m^−2^ (q3 weeks)	Placebo
Evaluable	29	50	150	43
Clinical complete response (CR)	29 (100%)	44 (88%)	15 (10%)	21 (49)
Clinical partial response (PR)	0 (0%)	6 (12%)	16 (11%)	7 (16%)
Clinical stable disease	0 (0%)	0 (0%)	60 (40%)	7 (16%)
Clinical progressive disease	0 (0%)	0 (0%)	59 (39%)	8 (19%)
All clinical responses (CR + PR)	24 (100%)	50 (100%)	31 (21%)	28 (65%)
Reference	(31, 32)	(37)	(38)	(39)
Cisplatin dose (frequency)	40 mg m^−2^ (x1 weekly)	40 mg m^−2^ (x1 weekly)		
Evaluable	22	238		
Metabolic complete response (mCR)	21 (95%)	173 (73%)		
Metabolic partial response (mPR)	1 (4%)	40 (17%)		
Metabolic stable disease	0 (0%)	0 (0%)		
Metabolic progressive disease	0 (0%)	25 (10%)		
All metabolic responses (mCR + mPR)	22 (100%)	213 (89%)		
Reference	(31, 32)	(40)		

*FDG PET, ^18^F-fluorodeoxyglucose positron emission tomography*.

*^a^Response Evaluation Criteria in Solid Tumors, version 1.0*.

For this article, an overall survival analysis was based on a total of 15 deaths from among the 29 eligible patients with regionally advanced stage cervical or vaginal cancer. The median follow-up from date of first treatment to database lock, censoring observed deaths, was 73 months (range, 58–129 months). A total of 25 (86%) of 29 patients survived either disease-free (*n* = 14) or died from a variety of non-cancer causes (*n* = 11). The low number of deaths on these two trials means that overall survival estimates are underpowered to show meaningful differences between triapine–cisplatin–radiation and historical cisplatin–radiation controls. Thus, the data here should be treated as preliminary. Overall survival estimates after triapine–cisplatin–radiation treatment was 83% (95% CI, 63–92%) at 30 months and 59% (95% CI, 39–74%) at 60 months (5 years). These two times were arbitrarily selected to match reporting in long-term analyses of cisplatin–radiation phase III trials in a similarly recruited cervical cancer patient population. In the comparator, estimates for overall survival after cisplatin–radiation treatment were 70% for 30 months and 60% for 60 months (5 years) (1). In an interesting subset of the 29-patient cohort analyzed here, 15 (52% of 29) patients at diagnosis had FDG PET-avid pelvic lymph nodes. Their overall survival after triapine–cisplatin–radiation combination treatment was 80% (95% CI, 50–93%) at 30 months and 60% (95% CI, 32–80%) at 48 months (4 years). In another study, estimated overall survival after cisplatin–radiation treatment in patients with FDG PET-avid pelvic lymph nodes was 50% at 30 months and 45% at 48 months (4 years) (41). CTEP regards this latter finding quite remarkable.

## Potential for Triapine in Cervical Cancer

In these two trials, triapine–cisplatin–radiation was a safe therapeutic investigational option for patients with untreated regionally advanced stage cervical or vaginal cancers. Patients had improvement in pelvic disease response and overall survival, suggesting pelvic disease control lowered the risk of cancer-related death.

Immediate adverse events related to triapine–cisplatin–radiation were mild and manageable in both trials. The trials did find an elevated rate of grade 3 or 4 platelet count decrease (20%). Cisplatin causes platelet apoptosis (42), possibly mediated by mitochondrial DNA depletion and apoptosis (43). Triapine could possibly exacerbate this effect. In a previous cisplatin–radiation trial, grade 3 or 4 platelet count decreases were seen in 1% of patients (44). The observed 16% rate of grade 3 or 4 leukopenia in these trials was similar to a 13% rate seen elsewhere (44). Rare late gastrointestinal or urological adverse events here were similar to prior trials. The limited adverse event profile demonstrated in these two trials suggests that there is further potential for combination clinical development.

Estimated survival was 83% at 30 months after triapine–cisplatin–radiation as upfront treatment for patients with regionally advanced stage cervical or vaginal cancer. This is better than the 70% rate at 30 months after cisplatin–radiation alone (1). But here patient survival must be interpreted cautiously, as sample sizes, and numbers of death events are both low in these two trials. It is known that up to half of primary cervix tumors retain residual microscopic cancer (2). This fits with an early cancer disease relapse pattern, as about 80% of disease relapses occur within 24 months after radiotherapy completion (1). In these two trials, triapine–cisplatin–radiation provided near total clinical and mCR (95%) in cervical or vaginal tumors. Many survived disease-free or died from other causes than cancer. In the absence of thorough microscopic examination after radical adjuvant surgery, it is impossible to determine triapine’s contribution to complete sterilization of residual microscopic disease. Here, FDG PET signal might serve as an unproven surrogate. A randomized trial better designed to test triapine’s contribution to clinical benefit is now active (clinicaltrials.gov, NCT02466971).

**Figure 2:**
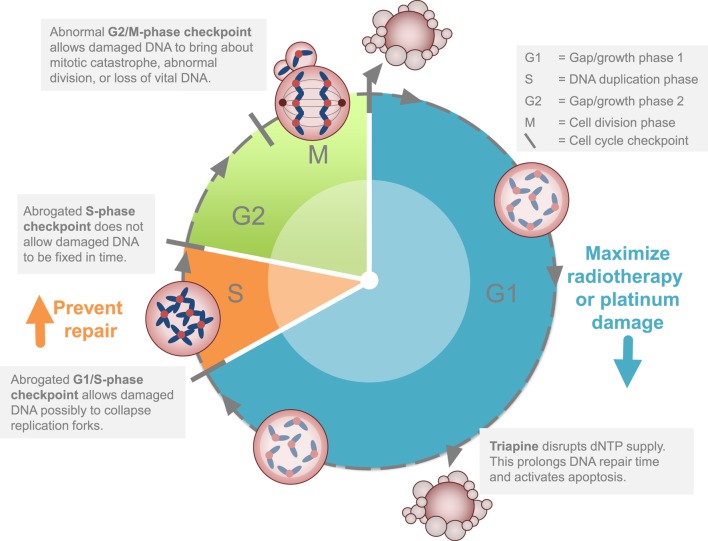
Strategy for ribonucleotide reductase (RNR) inhibitors in cervical or vaginal cancers. The clinical development strategy for RNR-targeted agents like triapine is to block or to stall DNA repair after a maximum amount of inflicted DNA damage occurs during the G1 or S phases of the cell cycle. This strategy is cytotoxic (1) by itself or (2) disrupts homologous recombination repair.

Two cell cycle concepts lie behind potential strategies targeting cancer DNA damage responses mediated at least in part by RNR activity (Figure 2). As outlined elsewhere (45), triapine increases susceptibility of cancers to G1-phase or S-phase-induced DNA damage. If the levels of G1-phase damage are sufficient, this can lead to cell death through replication catastrophe (46) or apoptosis (47). Cancer cell death may also happen if double-strand DNA damage carries through into mitosis, resulting in mitotic catastrophe (45). This would explain why radiation, which exerts its cytotoxic effect most powerfully in either the G1-phase or the G2-M-phase, pairs well with cisplatin, which manifests S-phase DNA damage. This would also explain why triapine might be so effective in combination with radiation–cisplatin. Sustained radiation–cisplatin DNA damage demands nucleotides for repair. Biologic agents like triapine render RNR small β subunits inactive and therefore places a much greater dependency on alternative nucleotide sources like deoxynucleoside salvage kinases. Sufficient DNA damage may be generated to exceed the threshold where cancer cells survive on alternative nucleotide sources, regardless of intact or exhausted cell cycle restriction checkpoints. RNR inhibitors that can maximize DNA damage, such as gemcitabine or hydroxyurea or triapine, provide means to overcome nucleotide supply as a vital cancer defense mechanism. In the two trials discussed here, evidence of improved disease response and patient survival advocate for this strategic attack on the cancer cell cycle.

In summary, these two trials provide response and survival evidence that triapine–cisplatin–radiation is effective in patients with regionally advanced stage cervical or vaginal cancer. Future studies should consider oral triapine for RNR blockade for patients in first-line cisplatin–radiation treatment.

## Ethics Statement

These trials were carried out in accordance with the recommendations of Case Western Reserve University and University Hospitals of Cleveland (Cleveland, OH, USA). All patients gave written informed consent in accordance with the Declaration of Helsinki. The Institutional Review Board of Case Western Reserve University and University Hospitals of Cleveland (Cleveland, OH, USA) approved both trial protocols.

## Author Contributions

CK and SI contributed to the collection and review of trial data, its analysis and authentication, and the writing and approval of this manuscript.

## Conflict of Interest Statement

CK and SI declare that the research was conducted in the absence of any commercial or financial relationships that could be construed as a potential conflict of interest.
